# Long-Term X-ray Findings in Patients With Coronavirus Disease-2019

**DOI:** 10.7759/cureus.15304

**Published:** 2021-05-28

**Authors:** Aarzoo Gupta, Ishan Garg, Abbas Iqbal, Abdul Subhan Talpur, Alyanna Marie B Mañego, Rama Kalyani Kavuri, Parkash Bachani, Sidra Naz, Zoya Qamar Iqbal

**Affiliations:** 1 Internal Medicine, Vardhman Mahavir Medical College and Safdarjung Hospital, Faridabad, IND; 2 Clinical Medicine, Ross University School of Medicine, Bridgetown, BRB; 3 Pediatrics, Ayub Teaching Hospital, Abottabad, PAK; 4 Medicine, Liaquat University of Medical and Health Sciences, Jamshoro, PAK; 5 Internal Medicine, SeriousMD, Muntinlupa, PHL; 6 Internal Medicine, Maratha Vidya Prasarak Samaj's Dr. Vasantrao Pawar Medical College Hospital and Research Centre, Nashik, IND; 7 Internal Medicine, Liaquat University of Medical and Health Sciences, Jamshoro, PAK; 8 Internal Medicine, University of Health Sciences (UHS), Lahore, PAK; 9 Internal Medicine, Jinnah Sindh Medical University, Karachi, PAK

**Keywords:** radiological finding, corona virus, covid-19, chest x-ray, long-term impact

## Abstract

Introduction: Reverse transcription-polymerase chain reaction (RT-PCR) and chest X-ray (CXR) are commonly used techniques for diagnosing and assessing prognosis in patients with coronavirus disease-2019 (COVID-19). This study aims to highlight the long-term radiological findings observed on CXR after recovery, in patients with COVID-19. This will help identify patients suffering from long-term consequences of COVID-19 and help them provide adequate care.

Methods: This study was conducted in the COVID-19 unit of a tertiary care hospital, Pakistan from August 2020 to February 2021. CXR of patients who were being discharged after negative PCR was done. Participants with positive X-ray findings, which included consolidation, reticular thickening, ground-glass opacities (GGO), pulmonary nodules, and pleural effusions, were enrolled in the study after getting informed consent. All findings were recorded in a self-structured questionnaire. Participants were scheduled to come for follow-up on day 30 after their initial CXR, where their CXR was repeated.

Result: Our results showed that n=429 (60.2%) participants had positive CXR at the time of discharge. After 30 days, n=371 participants returned for a follow-up X-ray. Out of the 371 participants, after 30 days, 123 participants still had positive CXR. Fatigue (41.4%) was the common symptom after 30 days. The most common finding was consolidation (82.1%), followed by reticular thickening (23.5%) on day 30.

Conclusion: In this study, although most of the patients completely recovered serologically from COVID-19, they still had radiological findings in their chest X-rays. Radiological findings are especially important in predicting the clinical course of the disease and may be used to monitor long-term complications.

## Introduction

The novel coronavirus, widely known as severe acute respiratory syndrome coronavirus 2 (SARS-CoV-2), has infected over 100 million humans worldwide. The first case of coronavirus disease-2019 (COVID-19) was recognized in Wuhan, China in December 2019 [1]. Due to its highly contagious nature, it quickly spread to other parts of the world, leading to the declaration of a pandemic by the World Health Organization (WHO) in March 2020 [2,3].

The symptoms of COVID-19 have been shown to range from mild fever, cough, headache, and fatigue to a more severe presentation with dyspnea, hemoptysis, and atypical pneumonia [4]. In the most severe cases, COVID-19 can lead to severe acute respiratory syndrome, respiratory failure, multi-organ failure with the resultant high mortality [4,5]. The diagnosis is confirmed by reverse transcription-polymerase chain reaction (RT-PCR) of samples obtained from oropharyngeal or nasopharyngeal mucosa and is supported by parenchymal changes seen on chest imaging [6,7]. The more common radiological changes noted on the chest imaging include ground-glass opacities (GGO) and consolidations while cavitation, pleural effusions, pleural thickening, bronchiectasis, and lymphadenopathy occur less frequently [8-10].

Improvement in the chest X-ray (CXR), along with negative PCR, has been seen to guide patient discharge in several studies [11]. Although RT-PCR and CXR are the more commonly used techniques for diagnosis, computed tomography (CT) scans of the chest have shown to have superiority in terms of higher sensitivity (98%) and can diagnose the disease even prior to symptom onset [12]. For this reason, most studies published in COVID-19 patients describe parenchymal changes seen on CT scans [9,10]. Limited studies have highlighted the role of CXR in monitoring disease course and in the follow-up of long-term changes in the lung parenchyma [7,13]. As CXR is the more readily available and cheaper imaging modality, it is essential that its role in disease progression and disease improvement should be known. Therefore, this study aims to highlight the long-term radiological findings observed on CXR after recovery, in patients with COVID-19.

## Materials and methods

This study was conducted in the COVID-19 unit of a tertiary care hospital, Pakistan from August 2020 to February 2021. CXR of patients who were being discharged after recovery was done and participants with positive X-ray findings were enrolled in the study after informed consent via consecutive convenient non-probability sampling. Recovery was defined as a negative PCR test for COVID-19. Positive CXR findings were consolidations, reticular thickening, GGO, pulmonary nodules, and pleural effusions. Ethical review board approval was taken.

Out of 712 participants, n=429 (60.25%) participants had positive CXR at the time of discharge. All findings were recorded in a self-structured questionnaire. The highest C-reactive protein (CRP) and lactate dehydrogenase (LDH) were recorded in questionnaires. Participants were scheduled for follow-up on day 30 after their initial chest X-ray, where their CXR was repeated. Fifty-eight (58) participants were lost to follow-up. Only participants who completed the study were included in the final analysis.

Statistical analysis was done using Statistical Package for the Social Sciences (SPSS) v. 22.0 (IBM Corporation, Armonk, New York, USA). Categorical data were presented as frequency and percentage. Numerical data were presented as mean and standard deviation.

## Results

Out of 712 participants, n=429 (60.2%) participants had positive CXR at the time of discharge. The most common symptom at the time of discharge was fatigue (77.3%). The most common finding was consolidation (78.9%), followed by reticular thickening (24.5%). In terms of location of findings, 66.5% of participants had bilateral radiological findings (Table 1).

**Table 1:** Symptoms, laboratory, and radiological findings on discharge. CRP: C-reactive protein, GGO: ground-glass opacities, LDH: lactate dehydrogenase, SOB: shortness of breath, mg/L: milligram per liter, IU: International Unit.

Characteristics	Frequency (percentages; n=371)
Age in years (mean ± SD)	42 ± 10
Gender	
Male	179 (48.2%)
Female	192 (51.8%)
Laboratory findings	
CRP (mg/L)	122.7 ± 21.4
LDH (IU)	328.1 ± 97.1
Symptom at discharge	
Fatigue	287 (77.3%)
Cough	101 (27.2%)
Dyspnea	61 (16.4%)
SOB	58 (15.6%)
Chest X-ray findings (location)	
Right	62 (16.7%)
Left	65 (17.5%)
Both	247 (66.5%)
Radiological findings	
Consolidation	293 (78.9%)
Reticular thickening	91 (24.5%)
GGO	81 (21.8%)
Pulmonary nodules	20 (5.3%)
Pleural effusions	18 (4.8%)

After 30 days, 371 participants returned for a follow-up X-ray. Out of 371 participants, on day 30, 123 (33.1%) participants still had positive CXR; 51 (41.4%) participants reported fatigue; 63.4% had bilateral radiological findings and the most common finding was consolidation (82.1%), followed by reticular thickening (23.5%; Table 2).

**Table 2:** Symptoms and radiological findings on 30-day follow-up GGO: ground-glass opacities, SOB: shortness of breath.

Characteristics	Frequency (percentages; n= 123)
Gender	
Male	63 (51.3%)
Female	60 (48.7%)
Symptom at 30 days	
Fatigue	51 (41.4%)
Cough	22 (17.8%)
SOB	15 (12.1%)
Dyspnea	18 (8.1%)
Chest X-ray findings at 30 days (location)	
Right	22 (17.8%)
Left	23 (18.6%)
Both	78 (63.4%)
Radiological findings	
Consolidation	101 (82.1%)
Reticular thickening	29 (23.5%)
GGO	20 (16.2%)
Pleural effusions	06 (4.8%)
Pulmonary nodules	05 (4.1%)

## Discussion

The result of our study demonstrated that 429 (60.9%) participants had positive CXR at the time of discharge. Among the symptoms, fatigue (77%) was found to be the most common one at the time of discharge. In terms of location of findings, 66.5% of participants had bilateral radiological findings. Consolidation (78.9%), followed by reticular thickening (24.5%) was the most common finding. 371 patients returned for a follow-up after 30 days, of which 123 (33.1%) participants still had positive CXR. After 30 days, 41.4% of the patients reported symptoms of fatigue, 63.4% had bilateral radiological findings and the most common finding was consolidation (82.1%), followed by reticular thickening (23.5%).

The results of our study are in line with other studies [2,14]. In concordance with our study, results from the systematic review of imaging findings conducted by Salehi et al. also showed the initial finding of CT scan in COVID-19 patients is bilateral, multilobar GGO with a peripheral or posterior distribution (or both), mainly in the lower lobes and less frequently within the right middle lobe [2]. Some studies also suggested that CT findings may vary among age groups, with consolidation more common in older groups and GGO in younger people [14]. The CT findings of COVID-19 patients in Korea were similar to those in China. However, the proportion of consolidation lesions were more common in Chinese people. On the basis of these radiological findings, Korean patients seem to experience a milder disease course than Chinese people [15]. In cases of long-term follow-up, studies have shown that approximately 98.1% of the chest CTs will show abnormalities even after 28 days of the onset of symptoms [16], and several case reports have demonstrated that post-discharge chest CTs show abnormalities but with slight improvements [17,18]. This has also been demonstrated in the findings of our study.

Even though the clinical and radiological course of COVID-19 is not predictable [19,20], there is proof that complications can exist for over a month. Even if patients do not show symptoms after being discharged for quite a few weeks or months, the idea of reinfection cannot go unnoticed. Additionally, abnormalities in the pulmonary function and radiological findings will exist and should be monitored for a long span of time. These could be more prominent in patients with pre-existing pulmonary or extrapulmonary diseases [21-23].

To the best of our knowledge, it is the first study from this region that highlights the long-term impact of COVID-19 on chest X-ray findings. However, since the study was conducted in a single institute, care should be taken while implying its result for a large population. Further large-scale studies are needed to identify the long-term CXR findings associated with COVID-19.

## Conclusions

In the case of COVID-19, keeping the long-term abnormal CXR in mind, follow-up visits after discharge are particularly important. They are just a check-up to observe changes in patient’s clinical conditions and ensure they are not any complications. It is also a great time to talk to a primary care provider about anything else or ask questions about mental wellbeing. The goal is to keep patients healthy and out of the hospital to prevent complications from happening again. Although most of the patients completely recovered from the disease, they are likely to have long-term lung damage. Radiological findings are particularly important in predicting the clinical course of the disease and may be used to monitor long-term complications.
